# Transcriptome Analysis and Metabolic Profiling of *Lycoris Radiata*

**DOI:** 10.3390/biology8030063

**Published:** 2019-08-29

**Authors:** Chang Ha Park, Hyeon Ji Yeo, Ye Eun Park, Seung-A Baek, Jae Kwang Kim, Sang Un Park

**Affiliations:** 1 Department of Crop Science, Chungnam National University, 99 Daehak-ro, Yuseong-gu, Daejeon 305-754, Korea; 2 Division of Life Sciences and Convergence Research Center for Insect Vectors, Incheon National University, Incheon 22012, Korea

**Keywords:** *Lycoris radiata*, transcriptome, metabolic profiling, galantamine biosynthesis

## Abstract

*Lycoris radiata* belongs to the Amaryllidaceae family and is a bulbous plant native to South Korea, China, and Japan. Galantamine, a representative alkaloid of Amaryllidaceae plants, including *L*. *radiata*, exhibits selective and dominant acetylcholinesterase inhibition. In spite of the economic and officinal importance of *L*. *radiata*, the molecular biological and biochemical information on *L*. *radiata* is relatively deficient. Therefore, this study provides functional information of *L*. *radiata*, describe galantamine biosynthesis in the various organs, and provide transcriptomic and metabolic datasets to support elucidation of galantamine biosynthesis pathway in future studies. The results of studies conducted in duplicate revealed the presence of a total of 325,609 and 404,019 unigenes, acquired from 9,913,869,968 and 10,162,653,038 raw reads, respectively, after trimming the raw reads using CutAdapt, assembly using Trinity package, and clustering using CD-Hit-EST. All of the assembled unigenes were aligned to the public databases, including National Center for Biotechnology Information (NCBI) non-redundant protein (NR) and nucleotide (Nt) database, SWISS-PROT (UniProt) protein sequence data bank, The Arabidopsis Information Resource (TAIR), the Swiss-Prot protein database, Gene Ontology (GO), and Clusters of Orthologous Groups (COG) database to predict potential genes and provide their functional information. Based on our transcriptome data and published literatures, eight full-length cDNA clones encoding *LrPAL2*, *LrPAL3*, *LrC4H2*, *LrC3H*, *LrTYDC2*, *LrNNR*, *LrN4OMT*, and *LrCYP96T* genes, involved in galantamine biosynthesis, were identified in *L*. *radiata*. In order to investigate galantamine biosynthesis in different plant parts of *L*. *radiata* grown in a growth chamber, gene expression levels were measured through quantitative real-time polymerase chain reaction (qRT-PCR) analysis using these identified genes and galantamine levels were quantified by high-performance liquid chromatography (HPLC) analysis. The qRT-PCR data revealed high expression levels of *LrNNR*, *LrN4OMT*, and *LrCYP96T* in the bulbs, and, as expected, we observed higher amounts of galantamine in the bulbs than in the root and leaves. Additionally, a total of 40 hydrophilic metabolites were detected in the different organs using gas-chromatography coupled with time-of-flight mass spectrometry. In particular, a strong positive correlation between galantamine and sucrose, which provides energy for the secondary metabolite biosynthesis, was observed.

## 1. Introduction

Members of the Amaryllidaceae family produce several alkaloids with unique structures and a variety of medicinal properties. Galantamine, in particular, is one of the alkaloids approved by the Food and Drug Administration (FDA), and the European Registration Bureau for treatment of Alzheimer’s disease. The low yield of galantamine from natural sources, and its high value in chemotherapy has encouraged researchers to elucidate the biosynthetic pathway of galantamine and other related alkaloids, as well as identify the key genes and enzymes in the pathway [1] to investigate their biological and chemical properties [2,3,4].

*Lycoris radiata*, which belongs to Amaryllidaceae family, is native to Northeast Asia including Korea, Japan, and China. Extensive research has been conducted in a several fields, including molecular biology [1,5], morphology [6], pharmacology [7], analytical chemistry [8], physiology [9], palynology [10], and chromosomal biology [11]. The plant is notable for its various biological activities, including anti-cancer, anti-malarial, anti-microbial, reduction in blood pressure, anti-inflammatory, cytotoxicity, and neuroprotective effects [12,13,14,15]. The biological properties are a result of the diverse secondary metabolites present in *L. radiata*. Studies have reported more than 20 alkaloids from this family that exhibit neuroprotective benefits [13,15,16]. Among the various alkaloids, 6β-acetoxycrinamine exhibited cytotoxicity in five human cancer cell lines [17]. The alkaloid lycorenine has anti-hypertensive effects [12], while the compound (±)5,6-dehydrolycorine is cytotoxic to astrocytoma and glioma cell lines, as well as exerts anti-malarial effects [13].

**Figure 1:**
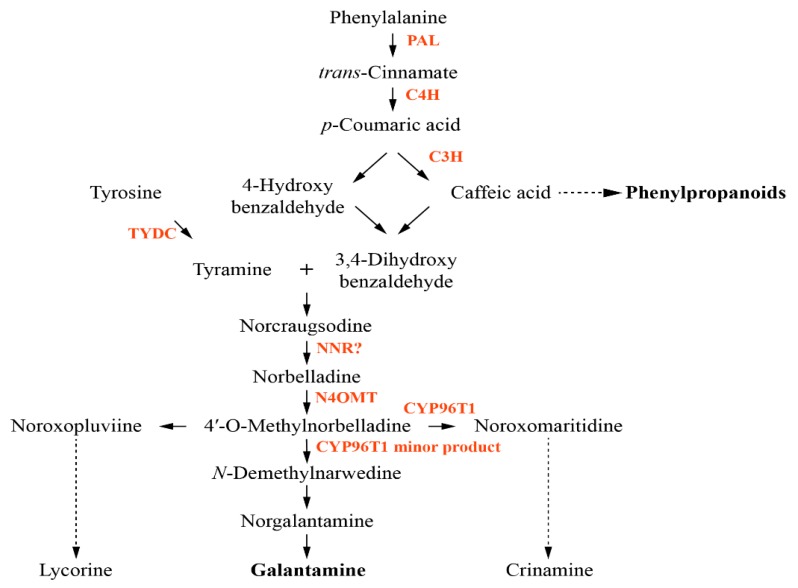
Proposed biosynthetic pathway for galantamine. Enzymes involved in the pathway are indicated in red. PAL, phenylalanine ammonia lyase; C4H, *trans*-cinnamate 4-monooxygenase; C3H, *p*-coumarate 3-hydroxylase; TYDC, tyrosine decarboxylase; NNR, noroxomaritidine/norcraugsodine reductase; N4OMT, norbelladine 4′-*O*-methyltransferase.

Galantamine is mainly obtained from the bulbs and flowers of *Galanthus caucasicus*, *Galanthus woronowii*, and other related genera such as *Narcissus pseudonarcissus* (*N. pseudonarcissus*), *Leucojum aestivum* (*L. aestivum*), and *L. radiata*. Galantamine is used to treat Alzheimer’s disease (AD) and as an AD painkiller [4]. Additionally, pretreatment with galantamine and nicotine modulates inflammation by attenuating tumor necrosis factor-alpha (TNF-α) and nitric oxide (NO) release through the nicotinic α7 acetylcholine receptors (α7 nAChR) and p44/42 mitogen-activated protein kinase (MAPK) pathway in murine microglia with nicotine [18]. The biosynthesis of galantamine, the genes and enzymes involved in the pathway are not well understood compared to other plant secondary metabolite pathways, including carotenoids and phenylpropanoids (Figure 1). Phenylalanine and tyrosine are formed from the shikimate pathway, and after synthesis of phenylalanine, firstly, phenylalanine ammonia lyase (PAL) catalyzes the conversion of phenylalanine to *trans*-cinnamic acid. Next, the hydroxylation of *trans*-cinnamic acid into *p*-coumaric acid is catalyzed by cinnamate-4-hydroxylase (C4H) [19,20]. The metabolite *p*-coumaric acid is potentially converted to caffeic acid by the *p*-coumarate 3-hydroxylase (C3H) enzyme [21]. Tyrosine decarboxylase (TYDC) catalyzes the conversion of tyrosine into tyramine [22]. The resulting compounds, 3,4-dihydroxybenzaldehyde and tyramine, are condensed to a Schiff-base and reduced to norbelladine by noroxomaritidine/norcraugsodine reductase (NNR) [19]. However, the precise role of NNR is unclear. Norbelladine is methylated by norbelladine 4′-*O*-methyltransferase (N4OMT) to form 4′-*O*-methylnorbelladine [23]. The resulting compound is used as an intermediate for the formation of the galantamine, lycorine, a toxic crystalline alkaloid present in many Amaryllidaceae plants [24], and crinamine, one of Amaryllidaceae alkaloids [25]. However, enzymes directly involved in galantamine formation have not been identified yet even though the involvement of cytochrome P450 enzymes (CYP96T1), involved in crinamine formation, has been discussed [26]. 

The transcriptome sequencing using next generation sequencing (NGS) technologies focuses on the transcripts containing functional information of the plant genome [27]. Metabolic profiling using gas-chromatography coupled with time-of-flight mass spectrometry (GC-TOFMS) is the measurement of low molecular weight metabolites and intermediates synthesized via a variety of metabolic pathways in plants [28]. To our knowledge, there are no studies on transcriptome analysis and metabolic profiling of *L. radiata*. Therefore, the aim of this study is to provide valuable data for a functional genomics resource and future biological research in *L. radiata* by characterizing the transcriptome and metabolome of *L. radiata* through the NGS-based and GC-TOFMS-based approach. Also, we provide information on galantamine biosynthesis in *L. radiata* in order to elucidate its pathway in future studies. 

## 2. Materials and Methods

### 2.1. Sample Preparation and RNA Extraction for Sequencing

Bulbs of *L. radiata* were washed with NaClO (4%, v/v) for 10 min and cut open to obtain growing point, and washed again with NaClO (4%, v/v) for 5 min and finally with NaClO (2%, v/v) for further 5 min. The bulbs were rinsed in sterilized distilled water and placed in plates containing half-strength MS medium mixed with 500 mg/L cefotaxime. The bulbs were incubated for 16 h in light and at 25 °C in a growth chamber. After a month, they were transferred to plates containing half-strength MS medium devoid of antibiotics. The clean bulbs of *L. radiata* were aseptically grown and used for RNA extraction. The aseptic plant parts, that is the bulb, leaf, and root were used for RNA extraction. Samples were collected in two biological replicates for the transcriptome analysis and Replicate I and Replicate II meant these two biological replicates. Total RNA was extracted from *L. radiata* using the cetyltrimethylammonium bromide (CTAB) method [29], and purified using the Total RNA Mini kit (Geneaid, Taiwan). The quality and quantity of total RNA was measured using a NanoVue Plus spectrophotometer (GE Healthcare, UK). Additionally, the RNA integrity was measured on a 1.0% denaturing agarose gel.

### 2.2. RNA Sequencing Using Illumina Platform

The procedure was carried out using our previously described method [30]. RNA sequencing was accomplished by using the TruSeq Stranded Total RNA LT Sample Prep Kit with Ribo-Zero Gold (Illumina, RS–122–2302, CA, USA). Ribosomal RNA from the total RNA was removed using biotinylated and target-specific oligos equipped with ribosomal RNA beads. The purified RNA was sheared at 94 °C for 8 min by using divalent cations. To isolate poly (A) mRNA, the oligo(dT)-Dynabead selection was used. Random hexamer primers, and reverse transcriptase. The cleaved RNA fragments were used as templates for the first strand cDNA synthesis. Remnant RNA was degraded using RNase H, and the second strand synthesis was initiated by incorporating dUTP instead of dTTP to form double-stranded cDNA. The cDNA was 3’-adenylated and subjected to adapter ligation. The last step involved the size selection of the resulting products through gel electrophoresis and PCR amplification to generate the final cDNA library. The library was sequenced on a paired-end (PE) flow cell using the NextSeq 500 sequencer (Illumina, CA, USA).

### 2.3. Pre-Processing of Raw Paired-End Reads

Both FastQC (https://www.bioinformatics.babraham.ac.uk/projects/fastqc/) and CutAdapt (http://pypi.python.org/pypi/cutadapt) software [31] were used to produce high-quality cleaned reads by eliminating low-quality reads, sequences containing unknown base N, adapter sequences, and reads with more than 10% *Q* < 20 bases, from the raw RNA sequencing reads to improve the de novo assembly process. After preprocessing, a total of 9,913,869,968 and 10,162,653,038 high-quality clean reads were obtained.

### 2.4. De Novo Assembly of L. Radiata Unigene Set

Trinity de novo assembly program (https://github.com/trinityrnaseq/trinityrnaseq/wiki) was used to reconstruct the full-length transcripts by organizing the overlapping reads as contigs without gaps [32]. The trimmed reads were assembled as contigs and the resulting contig properties, including average, maximum, minimum, median, and N50 length, were calculated using the Trinity software (https://github.com/trinityrnaseq/trinityrnaseq/wiki) [33]. The CD-HIT-EST software (http://weizhongli-lab.org/cd-hit/) was used to cluster the contigs based on sequence similarity [34]. The longest sequences were selected as unigenes. 

### 2.5. Annotation of L. Radiata Unigenes

BLASTN from the National Center for Biotechnology Information (NCBI) nucleotide non-redundant nucleotide sequence (nt) database (http://blast.ncbi.nlm.nih.gov/Blast.cgi) was used with a cutoff of E-value < 1.0 × 10^−5^. Six public databases (NCBI non-redundant protein (NR) database, SWISS-PROT (UniProt) protein sequence data bank, Brassica (BRAD) database, The Arabidopsis Information Resource (TAIR), and the Clusters of Orthologous Groups (COG) database) were used with the same cutoff value (E-value < 1.0 × 10^−5^) to identify protein coding sequences in the genomic DNA or proteins encoded by the transcripts. Based on SWISS-PROT annotation, unigenes of *L. radiata* were assigned gene ontology (GO) terms using the Blast2GO program (http://blast2go.com/b2ghome). The GO functional classifications were performed for all the unigenes, and distribution of gene functions at the macro level using WEGO software (http://wego.genomics.org.cn) was described [35]. The assembled transcripts were deposited in the National Center for Biotechnology Information (NCBI) Short Read Archive database (SRA, http://www.ncbi.nlm.nih.gov/Traces/sra/). The accession number is SRR8799443.

### 2.6. Identification of Genes Related to Galantamine Biosynthetic Pathways

Based on the transcriptome data, candidate genes involved in galantamine biosynthesis were identified and confirmed by either a nucleotide or a protein homology search in the NCBI GenBank database (http://www.ncbi.nlm.nih.gov/BLAST).

### 2.7. Plant Materials for Galantamine HPLC Analysis and Gene Expression Analysis

**Figure 2:**
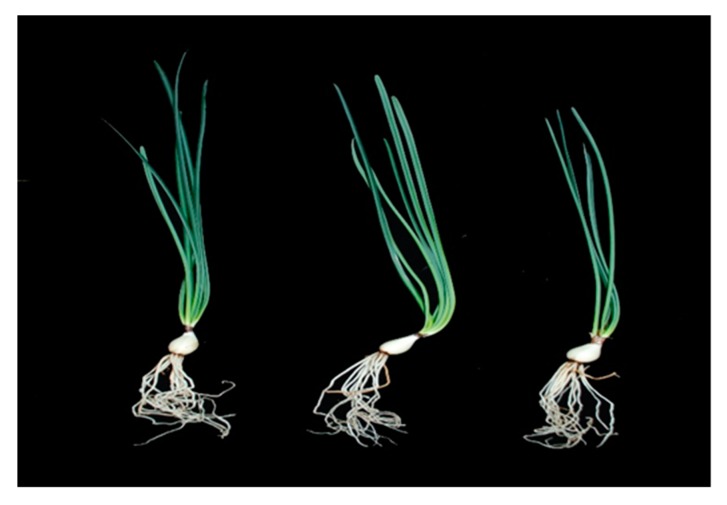
The phenotype of *L. radiata* grown in a growth chamber.

Bulbs of *L. radiata* were planted in 10 cm square pots and grown in a chamber (Figure 2). The photoperiod was 16 h light and 8 h darkness, and the chamber was maintained at 25 °C. The growth chamber was equipped with standard cool white fluorescent tubes with a flux rate of 35 μmol·s^−1^·m^−2^. After 3 months, the bulb, root, and leaf were harvested and powdered using liquid nitrogen for quantitative real-time polymerase chain reaction (qRT-PCR) of galantamine biosynthetic genes. Furthermore, the powdered samples were freeze-dried at −80 °C for 72 h for galantamine HPLC analysis. Three biological replicates were performed for each sample.

### 2.8. HPLC Analysis of Galantamine

Galantamine was extracted using a previously reported method with slight modification [36]. Fine powders (100 mg) of the various organs (leaf, bulb, and root) of *L. radiata* were weighed and 2 mL of 0.1% trifluoroacetic acid in water was added. After sonication for 30 min, the samples were left overnight at 4 °C. Next, the samples were sonicated for 30 min again and centrifuged at 13,000 rpm for 10 min. The supernatant was filtered into vials using a 0.45 µm Acrodisc syringe filter (Pall Corporation, Port Washington, NY, USA). Galantamine HPLC analysis was performed on a NS-4000 HPLC system coupled with a NS-6000 auto-sampler and UV-Vis detector (Futecs Corporation, Daejeon, Korea). An OptimaPak C18 column (250 × 4.6 mm, 5 μm, RStech Corporation, Daejeon, Korea) was used to separate galantamine using the mobile phase solvents composed of eluent (A) 50 mM ammonium formate aqueous buffer and eluent (B) acetonitrile at a flow rate of 1 mL/min. The column in the oven was thermostatically controlled to remain at 30 °C. The gradient program was set as follows: 0–15 min, 2% B; 15–30 min, 2–65% B; 30–31 min, 65–100% B; 31–35 min, 100% B; 35–36 min, 100%–2% B; and 36–38 min, 2% B (total time: 38 min). The detection wavelength was 285 nm, and 20 μL of the sample was injected for each run. Galantamine commercial standard was purchased (ChemFace, China). The calibration curve was plotted using six different known concentrations of galantamine. The linear equation was y = 12.7704 × −12.5000 (R^2^ = 0.9996). The values were represented as means ± standard deviation of three biological replicates.

### 2.9. qRT-PCR Analysis

Total RNA from the different organs (bulbs, leaves, and roots) was extracted using the CTAB method in combination with Plant RNA Mini Kit (Geneaid, Sijhih, Taiwan). The ReverTraAce^®^ Kit (TOYOBO, Osaka, Japan) was used for cDNA synthesis using the total RNA (1 μg) extracted from the different plant parts. Specific primers for genes involved in galantamine biosynthesis were designed using Primer3 (version 0.4.0, http://bioinfo.ut.ee/primer3-0.4.0/). The cDNA products were diluted 20-fold and subjected to PCR amplification in triplicate on a CFX96TM Real-Time System equipped with C1000^TM^ Thermal Cycler (Bio-Rad, USA) using the BioFACT™ 2× Real-Time PCR Master Mix kit with SFCgreen^®^ I (BioFACT, Daejeon, Korea). For each assay, 20 μL of qRT-PCR reaction mix consisted of 5 μL of the cDNA, 2 μL of 0.5 μM of gene-specific primer, and 10 μL of 2× Real-Time PCR Master Mix including SFCgreen^®^ I, and 3 μL of DEPC-treated water. The PCR reaction was carried out using the following thermal cycling conditions: Pre-denaturation at 95 °C for 15 min, 40 cycles of denaturation at 95 °C for 15 s, annealing at 55 °C for 30 s, and extension at 72 °C for 20 s. The run for each reaction consisted of a negative control filled with water instead of cDNA, and a series of standards. Three replications for samples of various plant parts were used for analysis using Bio-Rad CFX Manager 2.0 software (Bio-Rad, CA, USA). Based on the information provided in Table S1, primers were designed for use in real-time PCR experiments, which are listed in Table S2. The expression of galantamine biosynthesis genes was calculated using the 2^−∆Ct^ method [37]. The values were represented as means ± standard deviation of three biological replicates.

### 2.10. GC-TOFMS Analysis

The extraction of hydrophilic metabolites was carried out using a previously described method [38]. Fine powders (10 mg) of the various organs (leaf, bulb, and root) of *L. radiata* were weighed and extracted with 1 mL of a water/chloroform/methanol (1:1:2.5 v/v/v) mixture, followed by addition of 60 µL of adonitol (0.2 g L^−1^) as an internal standard. Next, a compact thermomixer was used for the extraction at a mixing frequency of 1200 × *g* and 37 °C for 30 min. After centrifuging the sample at 10,000 × *g* for 5 min, 800 µL of the polar phase was transferred to a new tube, to which 400 µL of deionized water was put. After centrifugation at 10,000 × *g* for 5 min, the methanol/water phase was evaporated in a CVE-2000 centrifugal concentrator for 3 h and the remaining material was then lyophilized using a freeze-dryer for 15 h. The lyophilized residues were subsequently processed in two stages: methoxime derivatization and trimethylsilyl etherification. Methoxyamine hydrochloride/pyridine (80 μL, 20 g L^−1^) was added to the vial, which was shaken at 30 °C for 90 min. After centrifuging to settle the solution to the bottom of the vial, 80 μL of *N*-methyl-*N*-(trimethylsilyl)trifluoroacetamide was added, followed by heating at 37 °C for 30 min. The resulting derivatized samples were analyzed using GC-TOF-MS in an Agilent 7890 A gas chromatograph (Agilent; Atlanta, GA, USA) that was equipped with a Pegasus HT TOF mass spectrometer (LECO, St. Joseph, MI, USA) and a CP-SIL 8 CB-MS column (30 m length, 0.25 mm diameter, 0.25 μm thickness; Varian Inc., Palo Alto, CA, USA). The injection, transfer line, and ion source temperatures were set to 230, 250, and 200 °C, respectively. The helium gas flow rate was 1 mL/min. The split ratio was set at 1:25. One-microliter (1 μL) samples were injected, and the oven temperature was maintained as follows: 80 °C for 2 min, ramping to 320 °C at a rate of 15 °C/min, then holding at 320 °C for 10 min. The mass scan range was 85–600 *m/z*, and the detector voltage was set at 1700 V. Quantification was performed using selected ions, and the Chroma-TOF software was used to locate peaks.

### 2.11. Statistical Analysis

The statistical difference among means was evaluated by Duncan’s multiple range test (DMRT) with a significance level of *p* < 0.05 using the Statistical Analysis System software (SAS, system 9.4, 2013; SAS Institute, Inc., Cary, NC, USA). Principal component analysis (PCA) was performed by the MetaboAnalyst 3.0 (http://www.metaboanalyst.ca/) with auto-scaling (unit-variance scaling). Also, the Pearson correlation analysis was performed using the SAS 9.4 and Multi-Experiment Viewer version 4.9.0 (http://www.tm4.org/mev/) was used for hierarchical clustering analysis (HCA) and heat map visualization. 

## 3. Results

### 3.1. Transcriptome Sequencing and De Novo Assembly of L. Radiata Sequences

The transcriptome analysis of *L. radiata* was performed in duplicate. All the raw reads (9,913,869,968 from replicate-1 and 10,162,653,038 from replicate-2) of *L. radiata* were obtained after trimming the adaptors and low-quality sequences with ambiguous N bases or more than 10% Q < 20 bases. The obtained reads were assembled de novo to contigs (233,502,958 and 284,508,455, respectively) with an average length of 447 bp and 441 bp, a maximum length of 19,408 bp and 17,939 bp, a median length of 312 bp and 314 bp, a minimum length of 224 bp, and N50 of 447 bp and 439 bp, respectively. The resulting contigs were assembled to unigenes (325,609 and 404,019, respectively) with an average length of 438 bp and 434 bp, a maximum length of 19,408 bp and 17,939 bp, a median length of 317 bp and 319 bp, a minimum length of 224 bp, and N50 of 434 bp and 431, respectively (Table 1). The unigene size distribution of *L. radiata* showed the following: Of the 325,609 and 404,019 unigenes, respectively, 80.35% (261,622) and 80.25% (324,226) were less than 500 nt; 13.78% (44,879) and 14.32% (57,857) were between 500 and 1000 nt; 5.56% (18,109) and 5.14% (20,783) were between 1000 and 3000 nt; 0.31% (999) and 0.25% (1153) were more than 3000 nt (Figure S1). 

### 3.2. Functional Annotation and Classification of L. Radiata Unigenes

The BLAST analysis of *L. radiata* unigenes (325,609 and 404,019, respectively) was performed against public databases (NR, Nt, SWISS-PROT, TAIR, COG and GO) with an E-value cutoff < 10^−5^). Specifically, a total of 120,043 unigenes (36.87% of all 325,609 unigenes) and 136,649 (33.82% of all 404,019 unigenes), respectively, were aligned in the databases, including 82,263 unigenes (25.26%) and 91,717 unigenes (22.70%) in the NR, 21,257 unigenes (6.53%) and 22,961 unigenes (5.68%) in the Nt, 53,960 (16.57%) and 60,706 (15.03%) in the SWISS-PROT, 59,051 (18.14%) and 65,053 (16.10%) in the TAIR, 15,559 (4.78%) and 16,206 (4.01%) in the COG, and 53,213 (16.34%) and 59,892 (14.82%) in the GO (Table S3).

**Figure 3:**
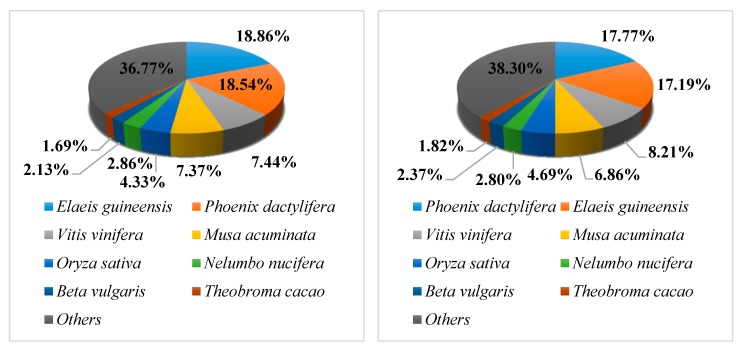
Species distribution of non-redundant (NR) annotation results of the *L. radiata* unigenes. Left, Replicate 1; Right, Replicate 2.

The E-value distribution revealed 25.16% and 22.34% of *L. radiata* unigenes, respectively, showing homology (E-value < 1.0 × 10^−60^), with 20.48% and 19.02% of the unigenes having high similarity (>80%) based on NR annotation (Figure S2A). However, the remaining 79.52% and 80.9% of the matched sequences revealed only a similarity between 18 and 80% (Figure S2B). For the species distribution, 18.86% of *L. radiata* unigenes of replicate 1 matched *Elaeis guineensis,* followed by *Phoenix dactylifera* (18.54%), *Vitis vinifera* (7.44%), *Musa acuminata* (7.37%), *Oryza sativa* (4.33%), *Nelumbo nucifera* (2.86%), *Beta vulgaris* (2.13%), *Theobroma cacao* (1.69%), and other plants (36.77%). Replicate 2 matched *Phoenix dactylifera* (17.77%) followed by *Elaeis guineensis* (18.86%), *Vitis vinifera* (8.21%), *Musa acuminata* (6.86%), *Oryza sativa* (4.69%), *Nelumbo nucifera* (2.80%), *Beta vulgaris* (2.37%), *Theobroma cacao* (1.82%), and other plants (38.30%), as shown in Figure 3.

The total number of the annotated unigenes were clustered into 26 functional categories based on the COG database. Of the 15,418 unigenes of replicate 1, 1699 unigenes (11.02%) were assigned to the category: translation, ribosomal structure, and biogenesis, representing the largest group of the 26 functional categories, followed by post-translational modification, protein turnover, chaperones (10.22%), carbohydrate transport and metabolism (9.41%), general functional prediction only (7.85%), signal transduction mechanisms (7.52%), and amino acid transport and metabolism (6.49%). Of the 16,072 unigenes of replicate 2, 1776 unigenes (11.05%) were assigned to the category: translation, ribosomal structure, and biogenesis, representing the largest group of the 26 functional categories, followed by post-translational modification, protein turnover, chaperones (9.78%), carbohydrate transport and metabolism (9.52%), general functional prediction only (7.76%), signal transduction mechanisms (7.58%), and amino acid transport and metabolism (6.46%), as shown in Figure S3.

**Figure 4:**
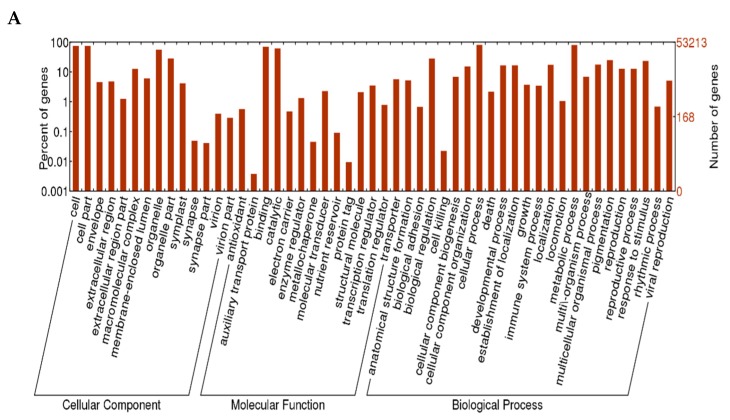

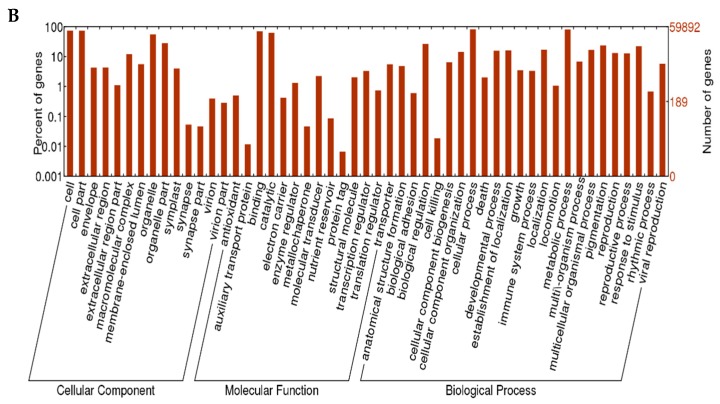
Gene Ontology (GO) annotation of the *L. radiata* unigenes. (**A**) replicate 1; (**B**) replicate 2.

Based on the proteins they encoded, the unigenes of *L*. *radiata* were clustered into three major categories: biological processes, cellular components, and molecular functions, with 51 functional sections as seen in Figure 4. A large number of unigenes (203,157 and 224,813 from replicates 1 and 2, respectively) were categorized in the biological process cluster, followed by the cellular component cluster, and the molecular function cluster. With respect to the biological process category, the unigenes were assigned to 23 functional sections. The majority of unigenes were involved in cellular processes. Among the unigenes clustered as the cellular component with 14 sections, most genes were associated with the cell and cell parts. Furthermore, 78,017 and 88,817 unigenes, respectively, matched the molecular function cluster with 14 sections, of which most unigenes were related to binding and catalytic activity.

### 3.3. Analysis of Galantamine Biosynthesis Genes in the L. Radiata Unigenes

Candidate genes for galantamine biosynthesis, based on data from the *L. radiata* transcriptome, were selected based on BLAST homology search. Analysis revealed a high degree of identity to other orthologous genes and proteins from various plant species. Thus, the genes were designated as *LrPAL2*, *LrPAL3*, *LrC4H2*, *LrC3H*, *LrTYDC2*, *LrNNR*, *LrN4OMT*, and *LrCYP96T* as described in Table S1.

### 3.4. Analysis of Galantamine Biosynthesis Genes

**Figure 5:**
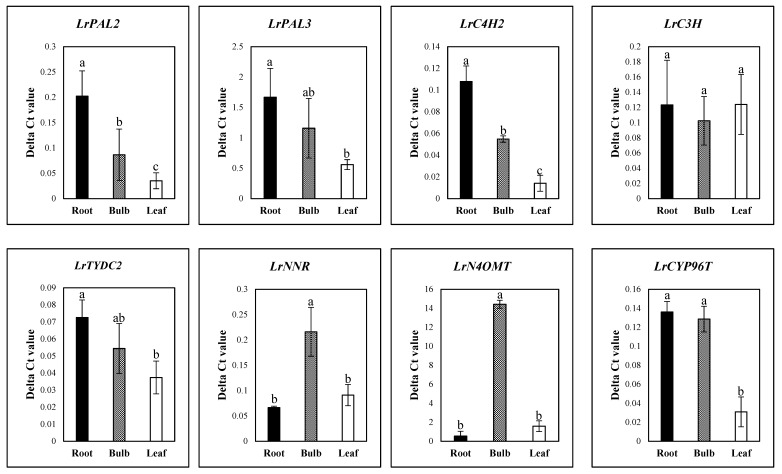
Expression of galantamine biosynthesis genes in different organs of *L. radiata* grown in a growth chamber. Different letters (a, ab, and b) indicate significant difference at *p* < 0.05. Duncan’s multiple range test (DMRT) was used.

Candidate genes for galantamine biosynthesis were used to design primers for qRT-PCR. qRT-PCR was performed with *LrActin* as a reference gene for normalization. The qRT-PCR results reveal the expression of *LrPAL2* and *LrC4H2* at an early stage in the pathway. Interestingly, the expression of these genes was significantly higher in roots. However, the expression levels of *LrNNR* and *LrN4OMT*, which are involved in galantamine biosynthesis, were significantly higher in bulbs than leaves and roots. The expression levels of *LrPAL2*, *LrTYDC2*, *LrC3H*, and *LrCYP76T* were not significantly different among the different parts of the plants tested in Figure 5.

### 3.5. Galantamine Content

HPLC analysis confirmed the presence of galantamine in all the organs (Table 2), including the root (0.53 ± 0.07 mg/g dry weight), bulb (0.27 ± 0.04 mg/g dry weight), and leaf (0.75 ± 0.09 mg/g dry weight). The galantamine level in the bulb was 1.42 and 2.78 times higher than that in the root and leaf, respectively.

### 3.6. Metabolite-Specific Profiling

**Figure 6:**
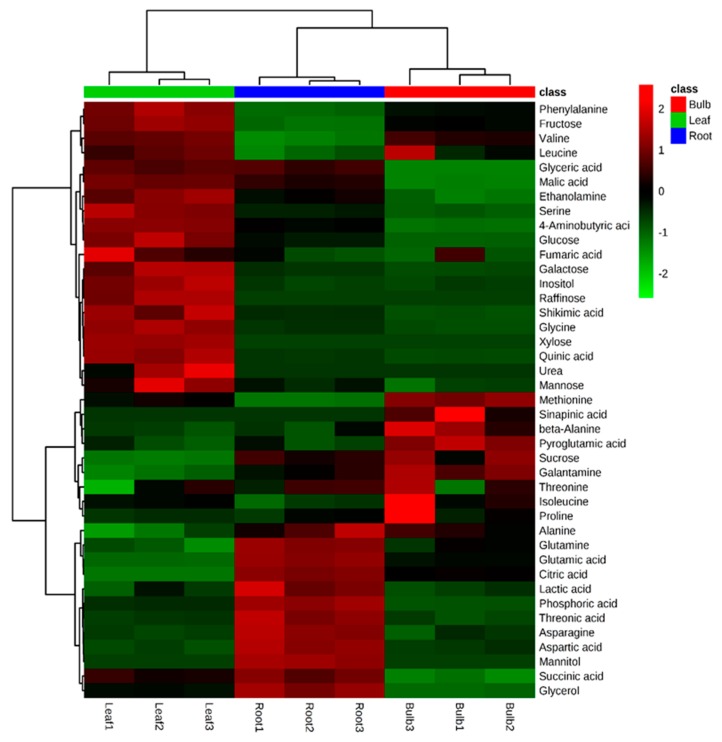
Heatmap representing differences in relative metabolite concentrations of *L. radiata* grown in a growth chamber. Increasing and decreasing the contents of metabolites are shown by red and green color, respectively.

A total of 40 metabolites were identified and quantified in the different organs of *L. radiata* using GC-TOFMS. However, urea, xylose, and raffinose were only detected in the leaf, sinapinic acid was only detected in the bulb, and mannitol was only present in the root (Figure 6). Specifically, most carbohydrates, with the exception of mannitol, sucrose, and raffinose, were higher in the leaf. However, sucrose is significantly higher in the bulb. Fourteen proteinogenic and three non-proteinogenic amino acids were detected and their levels varied between the different parts. In particular, the root contained higher levels of glutamine and asparagine, supported by the higher level of glutamic acid and aspartic acid, respectively. In contrast, serine was higher in the leaf, as explained by the higher level of glycine. Furthermore, the higher level of phenylalanine, derived from shikimic acid pathway, was supported by the higher level of shikimic acid. Among four intermediates from the tricarboxylic acid (TCA) cycle, the root contained the higher level of citric acid and succinic acid, associated with the higher levels of glutamic acid and glutamine (Table S4). 

The quantitative data was subjected to PCA to determine similarity or dissimilarity in metabolite profiles between the different parts of *L. radiata* (Figure S5). The two highest-ranking components explained 89.19% of total variance (component 1, 53.87%; component 2, 35.32%). Remarkably, the first component differentiated the leaf from the other organs. This separation could be attributable to sugars, sugar alcohols, several amino acids, and galantamine, for which the corresponding loading scores were negative and positive, respectively. The significant metabolites of component 1 were citric acid, sucrose, alanine, glutamic acid, galantamine, and glutamine, for which the eigenvector values were −0.19567, −0.19087, −0.18459, −0.17762, −0.17457, and −0.17308, respectively, and xylose, raffinose, inositol, quinic acid, glycine, and galactose, for which the eigenvector values were 0.21197, 0.21095, 0.21066, 0.20931, 0.20844, and 0.20641, respectively. Thus, the PCA results indicate that the leaf had higher levels of sugars and sugar alcohols than those of the root and bulb and that galantamine was higher in the root and bulb. 

**Figure 7:**
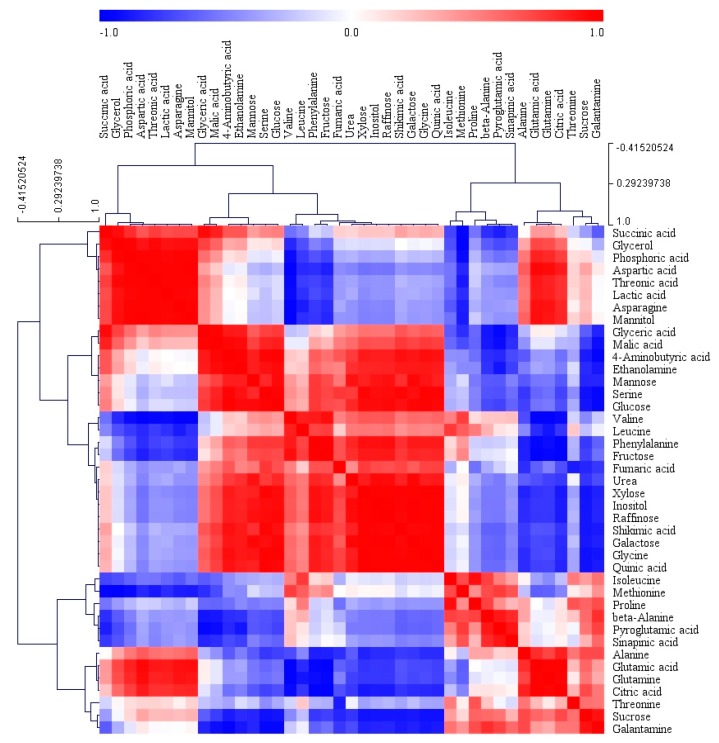
Correlation matrix and cluster analysis of results obtained from data on 41 metabolites for different organs of *L. radiata* grown in a growth chamber. Each square shows the Pearson’s correlation coefficient for a pair of metabolites, and the value for the correlation coefficient is represented by the intensity of the red or blue color as indicated on the color scale. Hierarchical clusters are represented by a cluster tree.

In order to gain insights into the relationships among the 41 metabolites identified in the different organs of *L. radiata*, a HCA using Pearson’s correlation results on the data sets was carried out (Figure 7). The results revealed the degree of correlation among the 41 metabolites. Glutamic acid and its derivatives, including glutamine, pyroglutamic acid, and proline, were positively correlated and citric acid was positively correlated with glutamic acid and its derivatives. There was a significantly strong relationship between aspartic acid and asparagine (*r* = 0.98637, *p* < 0.0001). Furthermore, a positive relationship was also detected between shikimic acid and phenylalanine (*r* = 0.80729, *p* = 0.0085). Among the identified sugars and sugar alcohols, mannose, glucose, fructose, xylose, inositol, raffinose, and galactose formed a group and showed a strong positive relationship. In particular, a significantly strong relationship was also found for sucrose and galantamine (*r* = 0.90876, *p* = 0.0007). These findings were consistent with the loading plot of PCA results. 

## 4. Discussion

RNA-Seq technology has been proven to be a valuable platform for transcriptomic studies of plants belonging to the Amaryllidaceae family, including *Lycoris aurea* (*L. aurea*) [39], *Allium cepa* [40], *N*. *pseudonarcissus* [41], and *Lycoris sprengeri* (*L*. *sprengeri*) [42]. In this study, clustering and assembly of clean reads produced 325,609 and 404,019 unigenes, respectively. Previous transcriptome studies showed a total of 141,111 unigenes of *L. aurea* [39] and 98,150 unigenes of *L. sprengeri* [42], respectively. Additionally, *L. radiata* unigenes had matches to *Phoenix dactylifera* and *Elaeis guineensis*, followed by *Vitis vinifera*, *Musa acuminate*, *Oryza sativa*, *Nelumbo nucifera*, *Beta vulgaris*, and *Theobroma cacao*.

Based on the GO annotation of *L. radiata* unigenes, the predicted unigenes were classified into three main groups, including molecular function, cellular component, and biological process. Most unigenes were associated with binding and catalytic activity in molecular functions, cell and cell parts in the cellular component, and finally, the cellular and metabolic processes. These results were consistent with the previous transcriptome study on *L. aurea*, in that most unigenes of *L. aurea* were also involved in “translation, ribosomal structure and biogenesis,” “post-translational modification, protein turnover, chaperones,” and “general functional prediction only,” and were also related to binding and catalytic activity, cell, cell parts, and organelle in the cellular component, and cellular and metabolic processes [39]. Furthermore, Chang et al. 2011 reported similar results for the GO category comparison with *L. sprengeri* transcriptome [42].

Galantamine is an alkaloid derived from phenylalanine and tyrosine and exhibits selective and dominant acetylcholinesterase inhibition. In this study, a total of eight galantamine-related genes were identified through the NCBI homology BLAST searches. The sequences of *LrPAL2, LrPAL3*, *LrC4H2*, *LrC3H*, *LrN4OMT*, and *LrCYP96T* genes were identical to those of *Narcissus papyraceus*. The *LrTYDC2* and *LrNNR* gene sequences, however, were related to *N. pseudonarcissus.* The results of qRT-PCR for the eight galantamine genes revealed relatively high levels of genes expressed, including *LrPAL2* and *LrC4H2* in the roots. However, in the bulbs, the levels of *LrNNR* and *LrN4OMT* were higher, which are important for galantamine biosynthesis. Furthermore, the higher expression of *LrNNR* and *LrN4OMT* and the high levels of *LrCYP96T* in the bulbs explained why bulbs contain higher amounts of galantamine than those of roots and leaves. These results are consistent with a previous study that reported that the bulbs of *Narcissus* sp. *aff*. *pseudonarcissus*, also one of the members of Amaryllidaceae family, contained very high amounts of galantamine and high level of *NpN4OMT* when compared to the leaves and inflorescence [23]. In this study, roots showed the lower level of galantamine than the bulbs although *LrPAL2* and *LrC4H2* were expressed at high levels in the root. In contrast, bulbs contained higher levels of galantamine and *LrN4OMT* and *LrNNR* expression. It suggests that the genes (*N4OMT* and *NNR*) expressed late in the galantamine biosynthesis pathway are more important than the genes (*PAL* and *C4H*) expressed early in the galantamine synthesis. Additionally, PAL and C4H are key enzymes in the phenylpropanoid biosynthetic pathway leading to the biosynthesis of various phenolic compounds (e.g., phenolic acid, flavonoid, anthocyanin, etc.,) [43]. The higher levels of *LrPAL2* and *LrC4H2* expression might provide intermediates used for the synthesis of various phenolics in the roots. However, further studies are needed to confirm this assumption. 

Sucrose has been used as a carbohydrate source for plant cultures since it is easily transported across membranes and metabolized by cells using sucrose transporters [44,45]. Furthermore, sucrose are involved in osmotic equilibrium [46] and can provide energy [47]. In this study, the bulb contained the highest levels of sucrose and galantamine. Specifically, bulbs and roots contained higher levels of sucrose than that of leaves. This might be because bulbs and roots are sugar sinks storing sucrose and leaves are sugar sources producing sucrose and transporting it to the sink organs. Similarly, Toit et al. 2004 reported that the bulbs and roots were the main storage organ of *Lachenalia* cv. Ronina while the inflorescence and leaves and were the main sugar source [48]. It was also reported that when sucrose is synthesized in leaves, as sugar sources, it is exported through the phloem to the other plant parts, thus, leaves does not have it for long [49]. A strong positive relationship between sucrose and galantamine was detected. This finding was consistent with several studies reporting that sucrose plays a significant role in the biosynthetic pathways of secondary metabolites, such as anthocyanin [50], phenolics [51], triterpenoids [52], and alkaloids [53]. In particular, the optimal concentrations of sucrose enhanced alkaloid and galantamine production in the shoot cultures of three Amaryllidaceae species (*N. pseudonarcissus*, *L. aestivum*, and *Galanthus elwesii*) [54]. Also, Selles et al. 1997 and Georgiev et al. 2009 reported that exogenous supply of sucrose induced enhanced accumulation of galantamine and alkaloids in shoot-clump cultures of *N. confusus* [55] and in shoot cultures of *L. aestivum*, respectively [56]. Li et al. 2018 reported that flowers of wild *L. radiata* in China contained high amounts of galantamine and had high expression of *LrC4H*, followed by leaf, bulb, and root [57]. The level of galantamine was not significantly different in the different organs of *L. chinensis*, but the root-hairs and bulbs contained amounts higher than those in the leaves [58]. Furthermore, monthly fluctuations in galantamine content in the bulbs of *L. aurea* was observed. Galantamine content slowly decreased from May to January, but the levels gradually increased from January to March, then begun to decrease [59]. Miaohua et al. 2012 reported the effect of light on galantamine content in the bulbs of *L. aurea.* They described that the galantamine content was the highest under 50% shading compared with that under 100% natural light [60]. Recently, Petruczynik et al. 2016 reported the analysis of galantamine in various member of Amaryllidaceae family. They found high amounts of galantamine in the leaves, followed by bulbs and roots of *Leucojum aestivum*; the highest amounts in roots, followed by leaves and bulbs of *Leucojum vernum*; the highest amount in the leaves, followed by roots and bulbs of *Leucojum vernum* var. *carpaticum*; the highest amount in roots, followed by bulbs and leaves of *Galanthus nivalis* [61]. Such variations in galantamine content are not surprising, as numerous factors are at play. It may be due to species specificity, climatic (annual average temperature and sunshine duration, altitude, latitude, and longitude) [62], or environmental factors (temperature, water availability, light intensity and availability, soil fertility, feeding by herbivorous insects and animals, interactions with parasites and pathogens, competition with neighboring plants) [63], which affect the quantity and quality of the plant derived compounds. Moreover, differences in the extraction methods, solvents, and analysis conditions lead to variations in the chemical composition [64,65].

## 5. Conclusions

This transcriptome study provides valuable new data for a functional genomic and further biological studies in *L. radiata*. In particular, the transcriptomic and galantamine analysis of *L. radiata* will provide useful information to elucidate the effect of various factors, including climatic and environmental factors, on galantamine biosynthesis. This study highlights the use of the GC-TOFMS-based metabolic profiling and the RNA-Seq for transcriptome research of *L. radiata*. Based on metabolic profiling and transcriptome data, a better understanding of the galantamine biosynthesis and relationship between primary metabolites and galamtamine was achieved. The information obtained from this study on galantamine biosynthesis and its distribution and composition will be helpful in developing potential therapeutics for AD.

## Figures and Tables

**Table 1:** Summary of transcriptome of the *L. radiata* unigenes.

Samples	Replicate I			Replicate II		
Sequencing Details	Raw Reads	Contigs	Unigenes	Raw Reads	Contigs	Unigenes
Total length (bp)	9,913,869,968	233,502,958	142,791,614	10,162,653,038	284,508,455	175,679,322
Number of sequences	6,5654,768	521,852	325,609	6,7302,338	644,408	404,019
Average length (bp)	151	447	438	151	441	434
Median length (bp)	151	312	317	151	314	319
Max length (bp)	151	194,08	19,408	151	17,939	17,939
Min length (bp)	151	224	224	151	224	224
N50 (bp)	151	447	434	151	439	431

**Table 2:** Galantamine content in different organs of *L. radiata* grown in a growth chamber.

Organs	Galantamine (mg/g Dry Weight)
Root	0.53 ± 0.07 b ^1^
Bulb	0.75 ± 0.09 a
Leaf	0.27 ± 0.04 c

^1^ Different letters (a, b, and c) indicate a significant difference at *p* < 0.05. Duncan’s multiple range test (DMRT) was used.

## Supplementary Materials

The following are available online at https://www.mdpi.com/2079-7737/8/3/63/s1, Figure S1: Length distribution of contigs and unigenes in the *L. radiata* transcriptome. (A) replicate 1, (B) replicate 2. Figure S2: Classification of NR annotation results of the L. radiata unigenes. Left, Replicate 1; Right, Replicate 2; (A) E-value distribution, (B) Similarity distribution. Figure S3: Clusters of Orthologous Groups (COG) function classification of the *L. radiata* unigenes. (A) RNA processing and modification, (B) chromatin structure and dynamics, (C) energy production and conversion, (D) cell cycle control, cell division, chromosome partitioning, (E) amino acid transport and metabolism, (F) nucleotide transport and metabolism, (G) carbohydrate transport and metabolism, (H) coenzyme transport and metabolism, (I) lipid transport and metabolism, (J) translation, ribosomal structure and biogenesis, (K) transcription, (L) replication, recombination and repair, (M) cell wall/membrane/envelope biogenesis, (N) cell motility, (O) post-translational modification, protein turnover, chaperones, (P) inorganic ion transport and metabolism, (Q) secondary metabolite biosynthesis, transport, and catabolism, (R) general functional prediction only, (S) function unknown, (T) signal transduction mechanisms, (U) intracellular trafficking, secretion, and vesicular transport, (V) defense mechanisms, (W) extracellular structures, (X) phage-derived proteins, transposases, and others, (Y) nuclear structure, and (Z) cytoskeleton. Figure S4: Principal component analysis results obtained from data on 40 metabolites for different organs of L. radiata grown in a growth chamber. Left, Score plot; Right, loading plot. PC 1, principal component 1; PC 2, principal component 2. Figure S5: The chromatogram of metabolites obtained from root (A), bulb (B), and leaf (C) of L. radiata by using GC-TOFMS. Peak identification: 1, lactic acid; 2, valine-1; 3, alanine; 4, valine-2; 5, urea; 6, serine-1; 7, ethanolamine; 8, phosphoric acid; 9, glycerol; 10, leucine; 11, isoleucine; 12, proline; 13, glycine; 14, succinic acid; 15, glyceric acid; 16, fumaric acid; 17, serine-2; 18, threonine; 19, β-alanine; 20, malic acid; 21, aspartic acid; 22, methionine; 23, pyroglutamic acid; 24, 4-aminobutyric acid; 25, threonic acid; 26, glutamic acid; 27, phenylalanine; 28, xylose; 29, asparagine; 30, glutamine; 31, shikimic acid; 32, citric acid; 33, quinic acid; 34, fructose-1; 35, fructose-2; 36, mannose; 37, galactose; 38, glucose-1; 39, glucose-2; 40, mannitol; 41, inositol; 42, tryptophan; 43, sucrose; 44, raffinose; IS, internal standard (adonitol). Table S1: Comparison of galantamine biosynthetic genes of L. radiata with the most orthologous genes and proteins. Table S2: Primers for genes in the galantamine biosynthesis pathway. Table S3: Summary of annotations of the L. radiata unigenes. Table S4: Metabolite peak height differences of root, bulb, and leaf of *L. radiata* using GC–TOFMS. N.D., not detected.
