# Supplementary material for: Transcriptome Analysis and Metabolic Profiling of Lycoris Radiata
Source: Biology (Basel). 2019 Aug 29;8(3):63. doi: 10.3390/biology8030063 (PMC6784096; doi:10.3390/biology8030063)
Supplement: Supplementary file 1 [file biology-08-00063-s001.pdf]

Supplementary Materials:

Transcriptome Analysis and Metabolic Profiling of *Lycoris Radiata*

Chang Ha Park, Hyeon Ji Yeo, Ye Eun Park, Seung-A Baek, Jae Kwang Kim * and Sang Un Park *

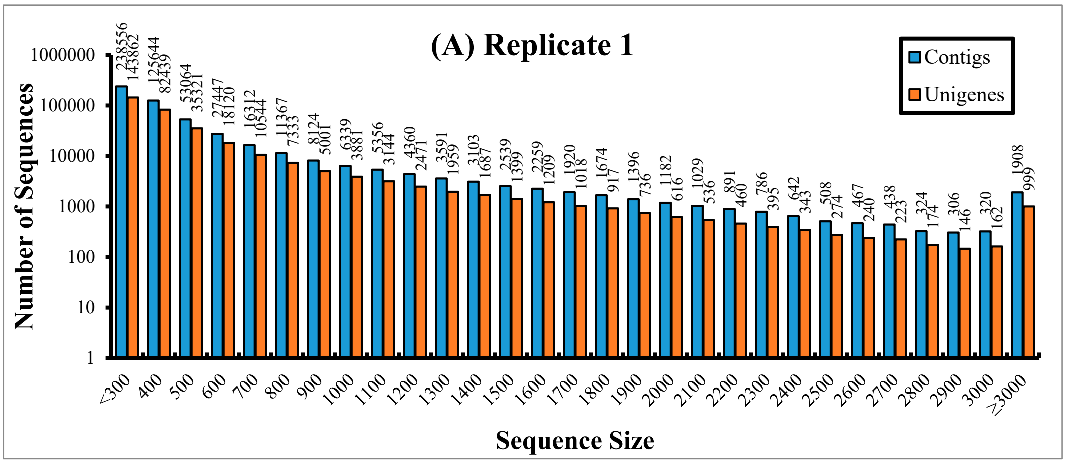

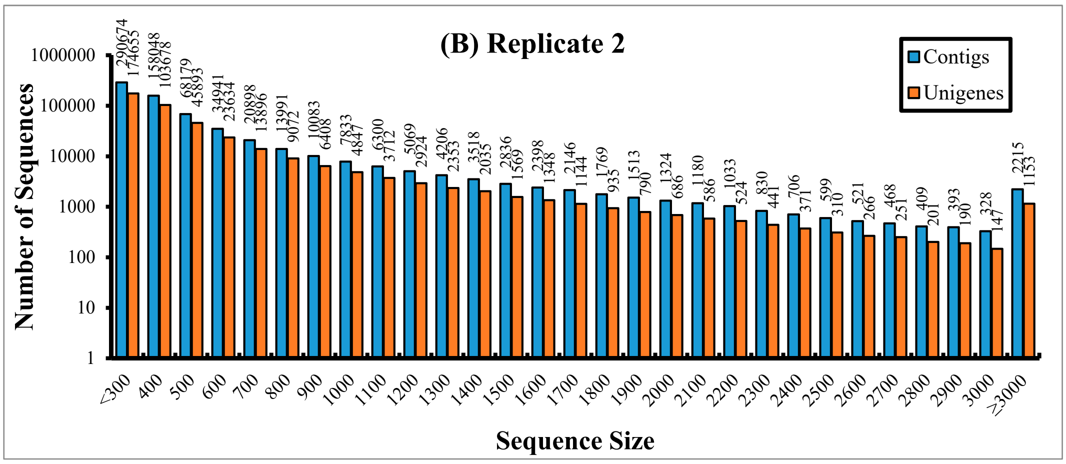

Figure S1. Length distribution of contigs and unigenes in the *L. radiata* transcriptome. (**A**) replicate 1, (**B**) replicate 2.

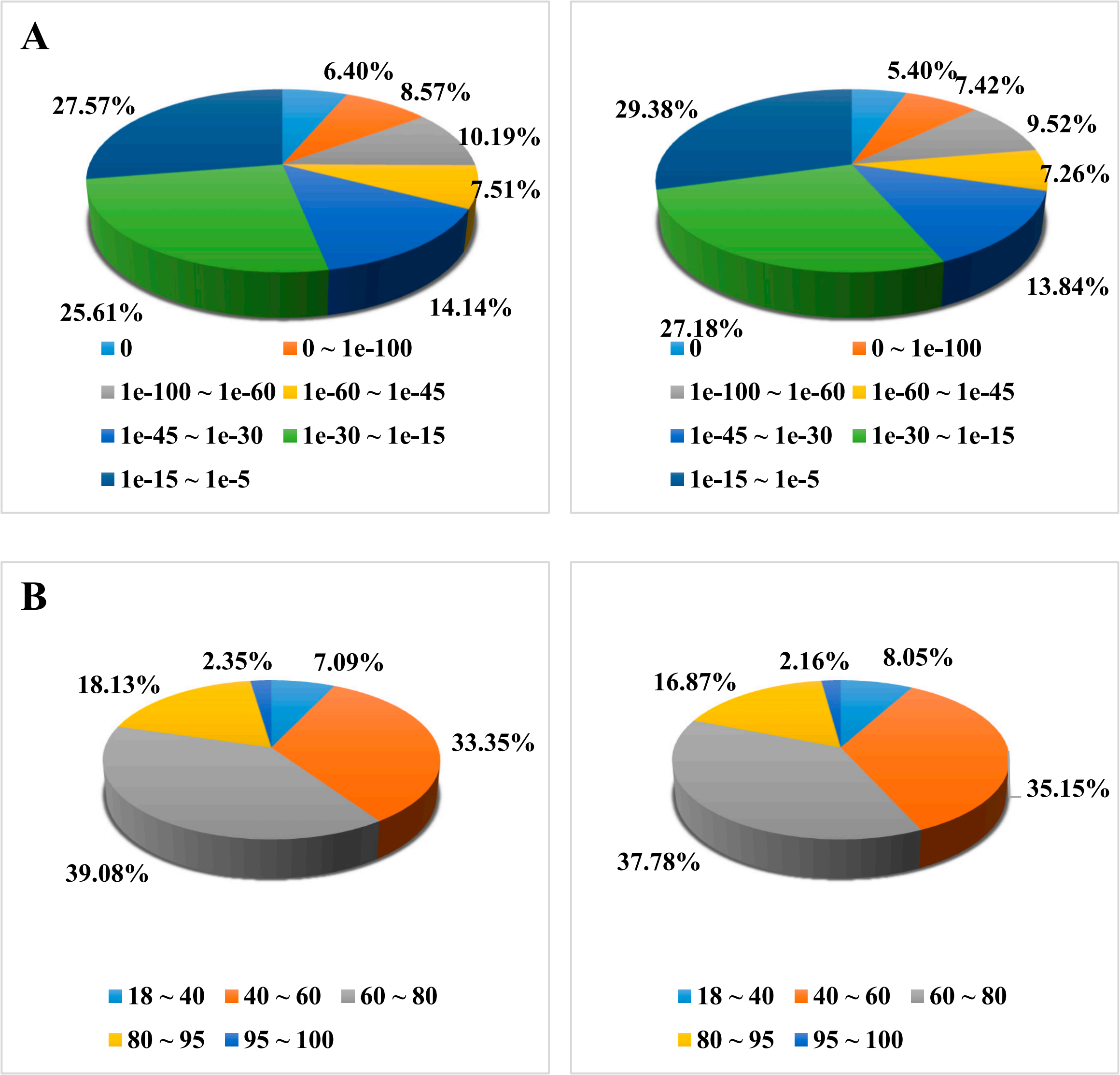

Figure S2. Classification of NR annotation results of the *L. radiata* unigenes. Left, Replicate 1; Right, Replicate 2; A. E-value distribution, B. Similarity distribution.

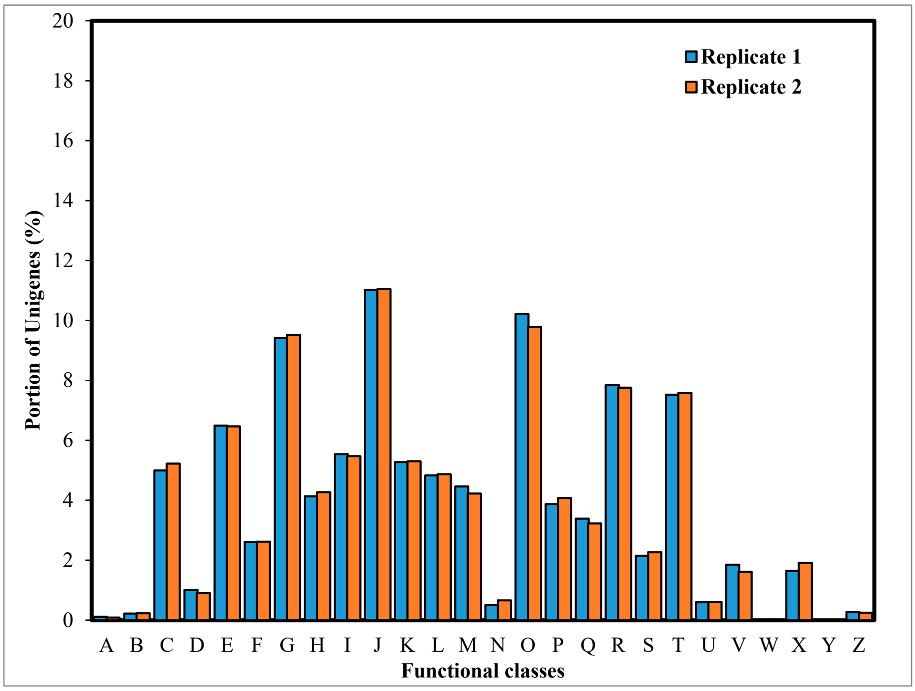

Figure S3. COG function classification of the *L. radiata* unigenes. A. RNA processing and modification, B. Chromatin Structure and dynamics, C. Energy production and conversion, D. Cell cycle control, cell division, chromosome partitioning, E. Amino acid transport and metabolism, F. Nucleotide transport and metabolism, G. Carbohydrate transport and metabolism, H. Coenzyme transport and metabolism, I. Lipid transport and metabolism, J. Translation, ribosomal structure and biogenesis, K. Transcription; L. Replication, recombination and repair, M. Cell wall/membrane/envelope biogenesis, N. Cell motility, O. Post-translational modification, protein turnover, chaperones, P. Inorganic ion transport and metabolism, Q. Secondary metabolite biosynthesis, transport and catabolism, R. General functional prediction only, S. Function unknown, T. Signal transduction mechanisms, U. Intracellular trafficking, secretion, and vesicular transport, V. Defense mechanisms, W. Extracellular structures, X. Phage-derived proteins, transposases and other, Y. Nuclear structure, and Z. Cytoskeleton.

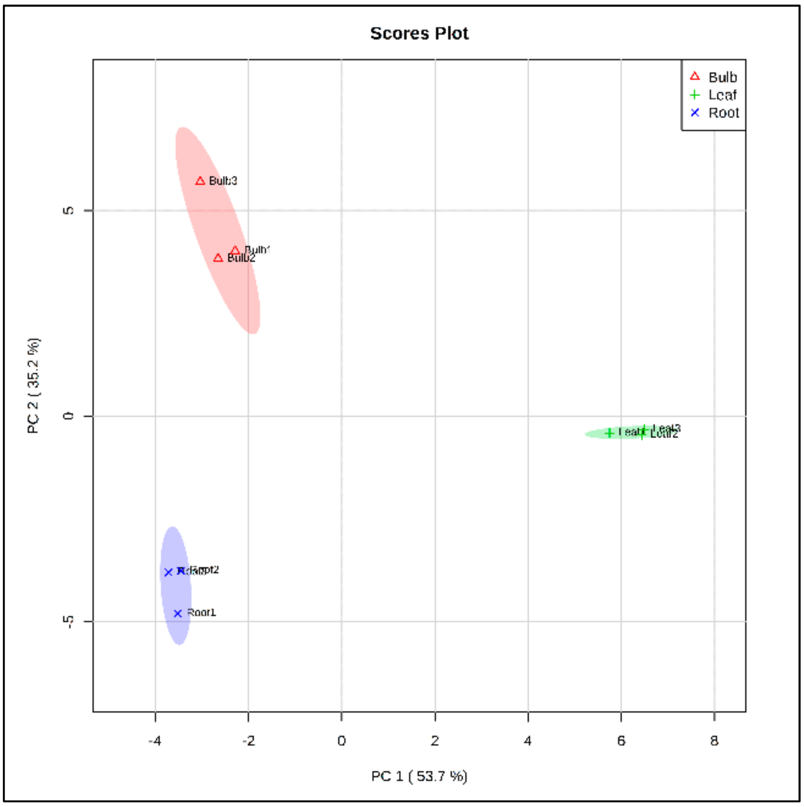

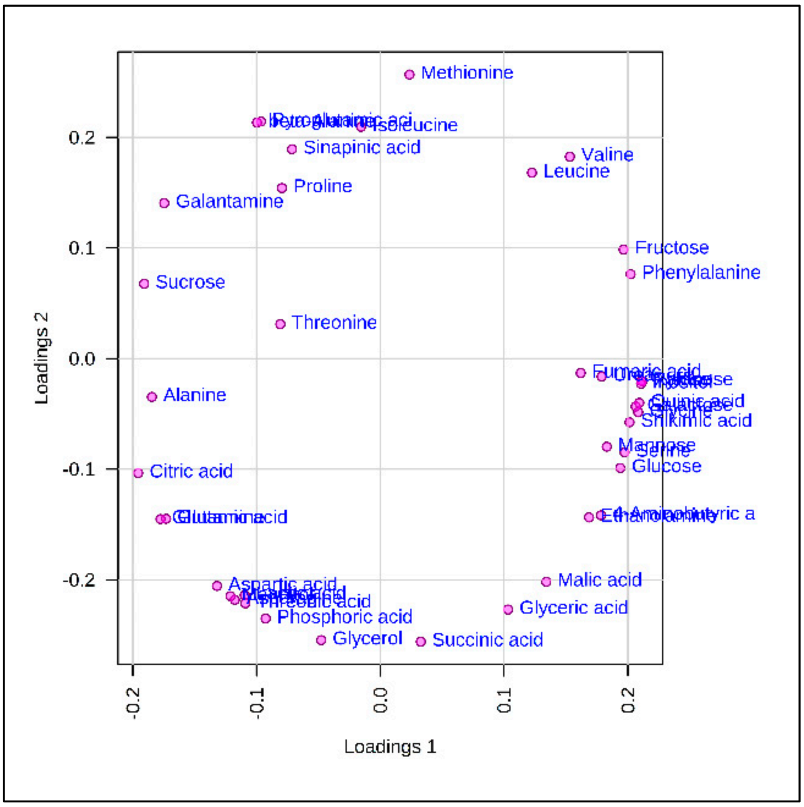

Figure S4. Principal component analysis results obtained from data on 40 metabolites for different organs of *L. radiata* grown in a growth chamber. Left, Score plot; Right, loading plot. PC 1, principal component 1; PC 2, principal component 2.

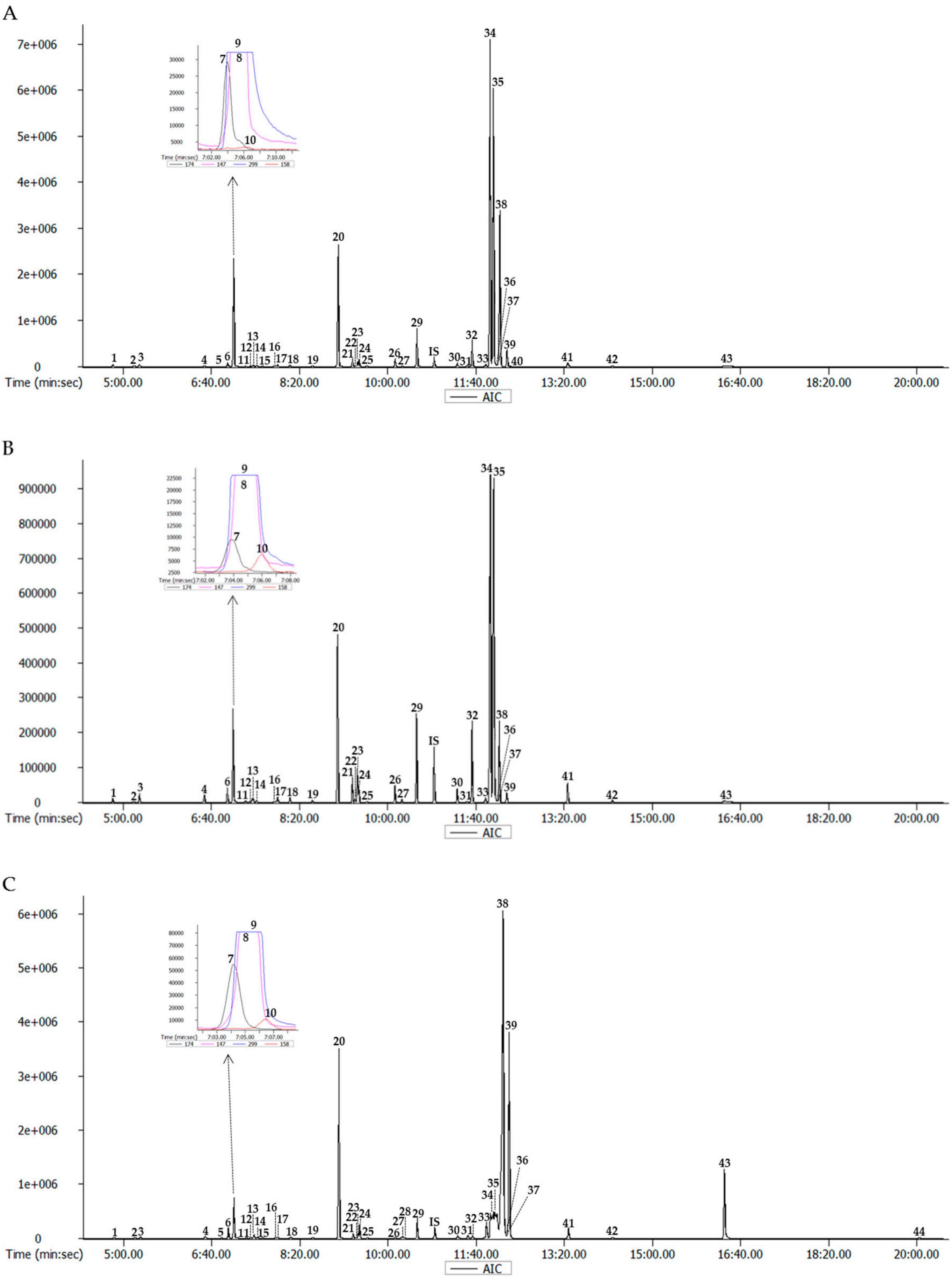

Figure S5. The chromatogram of metabolites obtained from root (A), bulb (B), and leaf (C) of *L. radiata* by using GC-TOFMS. Peak identification: 1, lactic acid; 2, valine-1; 3, alanine; 4, valine-2; 5, urea; 6, serine-1; 7, ethanolamine; 8, phosphoric acid; 9, glycerol; 10, leucine; 11, isoleucine; 12, proline; 13, glycine; 14, succinic acid; 15, glyceric acid; 16, fumaric acid; 17, serine-2; 18, threonine; 19, β-alanine; 20, malic acid; 21, aspartic acid; 22, methionine; 23, pyroglutamic acid; 24, 4-aminobutyric acid; 25, threonic acid; 26, glutamic acid; 27, phenylalanine; 28, xylose; 29, asparagine; 30, glutamine; 31, shikimic acid; 32, citric acid; 33, quinic acid; 34, fructose-1; 35, fructose-2; 36, mannose; 37, galactose; 38, glucose-1; 39, glucose-2; 40, mannitol; 41, inositol; 42, tryptophan; 43, sucrose; 44, raffinose; IS, internal standard (adonitol).

Table S1. Comparison of galantamine biosynthetic genes of L. radiata with the most orthologous genes and proteins.

Genes (Accession no.)	Plant species Orthologous genes (Accession no.)	Identity (%)	Plant species Orthologous proteins (Accession no.)	Identity (%)
LrPAL2 (MK690187)	*Narcissus papyraceus* (MF979857.1)	93.99	*Narcissus papyraceus* (AXU39893.1)	95.48
	Narcissus pseudonarcissus (MF405174.1)	93.29	*Narcissus pseudonarcissus* (AUG71935.1)	93.70
	Narcissus papyraceus (MF979858.1)	84.08	*Narcissus papyraceus* (AXU39894.1)	89.97
LrPAL3 (MK690188)	*Narcissus papyraceus* (MF979858.1)	92.80	*Narcissus papyraceus* (AXU39894.1)	94.77
	Narcissus pseudonarcissus (MF405174.1)	83.51	*Narcissus pseudonarcissus* (AUG71935.1)	90.34
	Narcissus papyraceus (MF979857.1)	82.52	*Narcissus papyraceus* (AXU39893.1)	89.83
LrC4H2 (MK690189)	*Narcissus papyraceus* (MF979860.1)	95.59	*Narcissus papyraceus* (AXU39896.1)	97.23
	Narcissus papyraceus (MF979859.1)	93.61	*Narcissus papyraceus* (AXU39895.1)	95.84
	Narcissus pseudonarcissus (MF416091.1)	92.68	*Narcissus pseudonarcissus* (AUG71936.1)	95.45
LrC3H (MK690190)	*Narcissus papyraceus* (KC455938.1)	96.12	*Narcissus papyraceus* (AXU39897.1)	97.53
	Narcissus pseudonarcissus (KP337736.1)	95.60	*Narcissus pseudonarcissus* (AUG71937.1)	95.74
	Narcissus tazetta cultivar Jinzhanyintai (AY987384.1)	93.36	*Narcissus tazetta* (AGI97941.1)	94.62
LrTYDC2 (MK690193)	*Narcissus pseudonarcissus* MF405172.1	98.00	*Narcissus pseudonarcissus* AUG71933.1	98.76
	Narcissus papyraceus MF979855.1	95.30	*Narcissus papyraceus* (AXU39891.1)	94.78
			Asparagus officinalis (XP_020265332.1)	83.83
LrNNR (MK690191)	*Narcissus aff. pseudonarcissus MK-2014* (KU295569.1)	84.42	*Narcissus pseudonarcissus* (AUG71944.1)	82.14
	Narcissus pseudonarcissus (MF416099.1)	84.16	*Narcissus papyraceus* (AXU39910.1)	73.02
	Narcissus papyraceus (MF979874.1)	78.27	*Narcissus papyraceus* (AXU39908.1)	70.63
LrN4OMT (MK690192)	*Narcissus papyraceus* (MF979869.1)	98.19	*Narcissus papyraceus* (AXU39905.1)	96
	Narcissus pseudonarcissus (MF416096.1)	96.39	*Narcissus* sp. *aff. pseudonarcissus MK-2014* (AIL54542.1)	95.82
	Narcissus sp. *aff. pseudonarcissus MK-2014* (KJ584561.1)	96.39	*Narcissus pseudonarcissus* (AUG71941.1)	95.40

LrCYP96T (MK690194)	*Narcissus papyraceus* (MF979870.1)	95.06	*Narcissus papyraceus* (AXU39906.1)	94.92
	Narcissus sp. *aff. pseudonarcissus* MK-2014 (KT693312.1)	93.41	*Narcissus* sp. *aff. pseudonarcissus* MK-2014 CYP96T2 (AMO65742.1)	93.37
	Narcissus pseudonarcissus (MF416098.1)	93.28	*Narcissus papyraceus* (AXU39907.1)	91.60

Table S2. Primers for genes in the galantamine biosynthesis pathway.

Genes	Sequence of forward primer (5′-3′)	Sequence of reverse primer (5′-3′)
PAL2	GGGCTACCATCAAATCTCTC	CATCTTGGTTGTGTTGTTCC
PAL3	GCAAACACTTCCATCTTCCAA	GCTCTTCTCTCACAAACCGA
C4H2	AGTCGCTTCTCGCCGTATTC	AGGTTGCGGTGGTTGAG
C3H	GACCACCCTCAACATCGT	GCAGACCTTCCTCACCTT
NNR	CGTTTGTGGAGGATAAGGA	AGTGATGTAGGAAGCAGATG
N4OMT	GCTGAGGGAGGTGACTGA	ATTGCCGTTATCTTTCCATCTTC
TYDC2	TTTGCCCAGAATACCGACA	AGAGCAGGGAGCAATCAAAG
CYP96T	CGATGCCGTGTCTTTCTAC	GGATTGCGTGTTTCTCTGC
ACTIN	AGGAATGGGTCAAAAGGATGC	TTGGCTTTCGGGTTCAGAG

Table S3. Summary of annotations of the *L. radiata* unigenes.

	Replicate I		Replicate II	
	BLASTed unigenes	Ratio (%)	BLASTed unigenes	Ratio (%)
All unigenes	325,609	100	404,019	100
Unigenes BLASTed against NR	82,263	25.26	91,717	22.7
Unigenes BLASTed against NT	21,257	6.53	22,961	5.68
Unigenes BLASTed against SWISS-PROT	53,960	16.57	60,706	15.03
Unigenes BLASTed against TAIR	59,051	18.14	65,053	16.1
Unigenes BLASTed against COG	15,559	4.78	16,206	4.01
Unigenes BLASTed against GO	53,213	16.34	59,892	14.82
Unigenes CDS predicted	63,816	19.6	70,096	17.35
All annotated coding unigenes	120,043	36.87	136,649	33.82

Table S4. Metabolite peak height differences of root, bulb, and leaf of *L. radiata* using GC–TOFMS. N.D., not detected.

.	Metabolites	Root	Bulb	Leaf
**Organic acid**	Lactic acid	17.29 ±2.45	8.17 ± 0.68	8.40 ± 1.68
	Urea	N.D.	N.D.	0.16 ± 0.09
	Phosphoric acid	1156.78 ± 42.88	168.48 ± 5.76	332.70 ± 16.92
	Threonic acid	7.04 ± 0.70	2.50 ± 0.26	2.81 ± 0.12
	Shikimic acid	7.46 ± 0.30	2.38 ± 0.42	33.70 ± 6.81
**Amino acid**	Valine	3.67 ± 0.75	15.01 ± 1.02	18.10 ± 0.78
	Alanine	17.86 ± 2.30	16.10 ± 1.08	11.62 ± 1.44
	Serine	57.92 ± 1.64	36.80 ± 1.75	117.28 ± 9.99
	Leucine	0.87 ± 0.41	3.01 ± 1.69	3.61 ± 0.36
	Isoleucine	1.00 ± 0.60	4.40 ± 2.93	2.25 ± 0.19

	Proline	0.69 ± 0.44	2.16 ± 2.27	0.33 ± 0.09
	Glycine	9.29 ± 0.28	6.65 ± 0.21	28.47 ± 1.38
	Threonine	7.91 ± 1.13	7.89 ± 2.83	6.33 ± 2.37
	beta-Alanine	2.90 ± 0.23	3.99 ± 0.49	2.78 ± 0.09
	Aspartic acid	49.28 ± 2.05	29.46 ± 0.74	27.83 ± 1.01
	Methionine	1.75 ± 0.07	6.41 ± 0.27	4.19 ± 0.45
	Pyroglutamic acid	42.09 ± 5.47	73.27 ± 5.57	40.25 ± 4.81
	4-Aminobutyric acid	63.97 ± 3.10	26.43 ± 1.15	105.99 ± 1.14
	Glutamic acid	71.91 ± 2.45	28.96 ± 1.62	2.90 ± 0.22
	Phenylalanine	6.29 ± 0.12	8.45 ± 0.11	12.02 ± 0.70
	Asparagine	357.61 ± 27.79	149.74 ± 28.00	150.52 ± 5.21
	Glutamine	32.34 ± 0.83	23.17 ± 2.26	17.44 ± 2.28
**Phenolic acid**	Quinic acid	21.15 ± 1.24	8.98 ± 0.69	153.58 ± 13.34
	Sinapinic acid	N.D.	0.48 ± 0.31	N.D.
**Photorespiration intermediates**	Glyceric acid	3.01 ± 0.24	0.00 ± 0.00	3.26 ± 0.14
	Ethanolamine	15.55 ± 2.03	5.10 ± 1.45	25.25 ± 2.88
**TCA cycle intermediates**	Succinic acid	9.38 ± 0.53	3.95 ± 0.32	7.82 ± 0.34
	Fumaric acid	1.39 ± 0.51	1.56 ± 1.10	3.36 ± 1.05
	Malic acid	1191.73 ± 46.05	297.32 ± 19.12	1492.63 ± 42.05
	Citric acid	251.19 ± 8.73	144.27 ± 5.10	20.72 ± 0.48
**Carbohydrates**	Xylose	N.D.	N.D.	0.20 ± 0.00
	Fructose	4348.55 ± 308.32	10144.22 ± 503.24	17026.43 ± 1165.01
	Mannose	4.16 ± 0.33	2.90 ±0.73	7.89 ± 2.20
	Galactose	3.34 ± 0.53	1.13 ± 0.30	23.53 ± 4.87
	Glucose	867.70 ± 80.44	129.20 ± 11.80	2479.09 ± 365.35
	Mannitol	1.88 ± 0.10	N.D.	N.D.
	Inositol	36.84 ± 2.03	36.61 ± 2.07	93.44 ± 9.19
	Sucrose	1745.01 ± 111.16	1996.55 ± 496.96	724.40 ± 20.03
	Raffinose	N.D.	N.D.	1.79 ± 0.27
	Glycerol	257.22 ± 15.59	69.23 ± 3.06	139.82 ± 4.09
